# Evaluation of optical coherence tomography angiography parameters in patients treated with Hydroxychloroquine

**DOI:** 10.1186/s12886-021-01977-5

**Published:** 2021-05-11

**Authors:** Mohammadreza Akhlaghi, Farzan Kianersi, Hamed Radmehr, Alireza Dehghani, Afsaneh Naderi Beni, Pegah Noorshargh

**Affiliations:** Isfahan Eye Research Center, Department of Ophthalmology, Isfahan University of Medical Science, Isfahan, Iran

**Keywords:** Optical coherence tomography angiography, Hydroxychloroquine, Vessel density, Foveal avascular zone

## Abstract

**Background:**

One of the major side effects of Hydroxychloroquine (HCQ) is retinopathy. The aim of this study was to evaluate the Optical coherence tomography angiography (OCTA) parameters in a group of patients who have Hydroxychloroquine-induced retinopathy based on Multifocal electroretinography (mfERG) with a group who do not have retinopathy.

**Method:**

This is a Cross-Sectional Study. In this study, patients with Rheumatoid arthritis (RA) or Systemic lupus erythematosus (SLE) who had been taking Hydroxychloroquine for at least 7 years were included. MfERG and OCTA imaging were performed for all patients. Patients were divided into Normal mfERG and Abnormal mfERG groups based on mfERG results. OCTA parameters were studied in these two groups.

**Result:**

Sixty-one patients (61 eyes) were included. Forty-one patients had SLE and 20 patients had RA. Forty patients (66.7%) had Abnormal mfERG. The mean vascular density (VD) in Superficial capillary plexus (SCP) layer was not significantly different between Normal mfERG and Abnormal mfERG groups (*P*-Value> 0.05). Mean VD in SCP layer was not significantly different between Normal mfERG and Abnormal mfERG groups (*P*-Value> 0.05). In RA subgroup, mean VD in SCP layer in PeriFovea region in Abnormal mfERG group was significantly lower than normal group (*P*-Value < 0.05). Mean VD in deep capillary plexus (DCP) layer in Whole Image, Superior Hemi, Inferior Hemi, PeriFovea area in Abnormal mfERG group was significantly lower than normal group (*P*-Value < 0.05). This discrepancy was also observed in the RA subgroup but not in the SLE subgroup. The mean of none of the parameters of foveal avascular zone (FAZ) (mm2), Flow Area of Outer Retina (mm2) and Flow Area of Choriocapillaris (mm2) were not statistically significant between the groups Abnormal mfERG and Normal mfERG. (*p*-value> 0.05).

**Conclusion:**

VD in the DCP layer decreased in abnormal mfERG patients compared to patients with normal mfERG. But it seems that VD in SCP layer, FAZ Area and Flow Area are similar in both groups. OCTA may be used as a non-invasive tool in the diagnosis of early stages of HCQ-induced retinopathy, especially in RA patients, but further studies are needed.

## Background

Hydroxychloroquine (HCQ) is an anti-malarial drug that is widely used in the treatment of rheumatic diseases including Systemic lupus erythematosus (SLE) and Rheumatoid arthritis (RA) [1]. An important ocular complication of HCQ is retinal toxicity [2–4], the mechanism of which is not well understood. HCQ is likely to bind to melanin within retinal pigment epithelium (RPE) cells, and its concentration in RPE cells increases over time and disrupts retinal cell metabolism and destroys photoreceptors and the Outer Nuclear Layer (ONL) [5–7].

HCQ-induced retinopathy is irreversible and can cause blindness, and may persist even after discontinuation of the drug [8, 9]. And if toxicity is detected before RPE cell damage occurs, it can maintain the patient’s central vision [10]. According to previous studies, the prevalence of this complication at doses less than 5 mg / kg / daily based on real body weight in the first 10 years of drug use is less than 2% [11–13]. However, in more than 10 years of use of the drug and the presence of kidney disease or concomitant use of tamoxifen, it reaches 20% [14].

To screen patients for HCQ-induced retinopathy as recommended by the American Association of Ophthalmology (AAO), all patients are screened at the start of medication, then annually after 5 years of medication, and individuals Which have a major Risk Factor are examined at shorter intervals. To diagnose HCQ-induced retinopathy, various methods have been recommended, including: Spectral Domain Optical Coherence Tomography (SD-OCT), automated perimetry test, multifocal electroretinogram (mfERG), FUNDUS AUTOFLUORESCENCE (FAF) IMAGING [15]. But it is controversial as to which of these methods is the Gold Standard early detection of HCQ-induced retinopathy [15–20]. So it’s important to find a tool that can help us diagnose early.

mfERG is highly sensitive among the mentioned tests and because it is an objective test, it is less dependent on the patient’s response and cooperation [15]. The mfERG objectively evaluates the electroretinographic response of the macular region, and in HCQ retinopathy, this response is reduced in the paracentral region [17].

Optical coherence tomography angiography (OCTA) is a new imaging technique without contrast injection that can show the vascular structure of the retina and choroid based on the flow of red blood cells and It is used to study the course and pathogenesis of retinal diseases such as Diabetic retinopathy, Choroidal neovascularization (CNV), Central retinal vein occlusion (CRVO) [21]. It may also be helpful in the early detection of HCQ-induced retinopathy. To the best of our knowledge, there are very few articles in this field [22, 23].

The aim of our study was to compare the quantitative and qualitative parameters of OCTA including vascular density, foveal avascular zone (FAZ) with MFERG changes in patients with a history of HCQ. We think this study may help to better understand the mechanism of HCQ-induced retinopathy and its early diagnosis.

## Method

This is a cross-sectional study performed in retina clinic of Feiz Hospital and Didavaran Eye Center, Isfahan, Iran in 2019–2020, Approved by the Clinical Research Ethics Committee of Isfahan University of Medical Sciences. Informed consent was obtained from all patients participating in the study.

All patients with a history of at least 7 years of Hydroxychloroquine due to Systemic lupus erythematosus (SLE) or rheumatoid arthritis (RA), who referred to the retinal clinic of Feiz Hospital during 2019–2020 were included in this study. One eye from each patient was included in the study, so that if the patient’s month of birth was even, the right eye and if the patient’s month of birth was odd, the patient’s left eye was included in the study. Patients with exclusion criteria were excluded from the study, including history of ocular surgery, history of ocular disease, comorbid systemic diseases (such as hypertension (HTN) and diabetes mellitus (DM) and vascular diseases), refractive error spherical equivalent greater than 4.00D, Intraocular pressure (IOP) above 21 mmHg, kidney disease or failure, concomitant use of tamoxifen and presence of maculopathy due to other diseases.

A complete medical and ophthalmological history was taken from all patients, including: demographic information, type of disease, duration of disease, drugs and their amount and duration of use. All patients underwent a complete ophthalmologic examination by a vitreo retinal fellowship, Includes: refraction, best corrected visual acuity (BCVA) (in decimal and LogMAR chart (Logarithm of the Minimum Angle of Resolution)), IOP, anterior segment examination and fundoscopy with dilated pupil. Spectral-domain OCT (SD-OCT), Optical coherence tomography angiography (OCT-A), multifocal electroretinogram (mfERG), Humphrey Visual Field Test 24–2, Fundus autofluorescence (FAF) were performed for all patients.

mfERG was performed by RETI Scan (Roland consulting company) and software 6.16.3.10 according to the protocol of the International Society for Clinical Electrophysiology of Vision (ISCEV) by an expert optometrist. The 61-hexagon strategy was used and the stimulus was calibrated according to the ISCEV guidelines. The mean amplitudes of N1, P1, N2 were analyzed in five concentric areas of the macula [24, 25].

To interpret mfERG in HCQ retinopathy screening, different criteria have been used in various articles, such as: amplitude reduction, implicit time extension, ring ratio and amplitude reduction based on Color Difference Plot [8, 25–27]. In this study, we considered abnormal values of R1 or R2 amplitude or Ring Ratio (R1 / R2, R1 / R3, R1 / R4, R1 / R5) in each of the patient’s eyes as abnormal mfERG. The normal range of mfERG parameters was determined based on the normal database of our center, which has been corrected based on age, sex.

OCTA imaging was performed with RTVue XR device (OPTOVUE, California, USA) and software version 2018.0.0.14. Macular imaging was performed with a 6 * 6 mm scans. The segmentation was done automatically and checked by an expert technician. All images were taken with dilated pupil and by the same machine, and in similar conditions at 8 A.M by an expert technician. And quantitative values of vascular density (in superficial retinal capillary plexus (SCP) and deep capillary plexus (DCP)), foveal avascular zone (FAZ), Flow Rate (in choriocapillaris, outer retina layers) and Retinal Thickness automatically by the software was calculated. Images with signal strength index (SSI) less than 40 or had an artifact were not analyzed [28]. Finally, based on mfERG criteria, we divided patients into normal mfERG and abnormal mfERG groups. We also divided patients into separate SLE and RA subgroups.

### Statistical analysis

Data were analyzed using the SPSS version 21.0 Statistical package (SPSS Inc., Chicago, IL, USA). Quantitative and qualitative data were presented as Mean ± SD, Median **[**maximum, minimum**]** and frequency (percentage). Normality of continuous data was evaluated using Kolmogorov-Smirnov test. Independent t-test was used for comparing data between groups. Qualitative data were compared between groups using chi-square test and fisher exact test. Pearson correlation were used to investigate the correlations between duration of the treatment, cumulative dose and Vessel Density, FAZ Area, Flow Area based. Statistical significance level was set at *P* < 0.05.

## Result

Sixty-one patients (61 eyes) with a history of at least 7 years of Hydroxychloroquine use due to SLE or RA were studied. Of these, 57 were female (93.4%) and 4 were male (6.5%) with a mean age of 46.9 ± 11.62 years. Forty patients (40 eyes) had SLE and 21 patients (21 eyes) had RA. In general, out of 61 patients (61 eyes), 40 (65.5%) were considered in the Abnormal mfERG group. Demographic and clinical variables of individuals are shown in Table 1. Patients did not have a statistically significant difference in any of the demographic and clinical variables including age, sex, mean daily dose of HCQ, total dose and BCVA (Best-corrected visual acuity) in normal and abnormal groups (*P*-VALUE> 0.05).

**Table 1:** The Demographic and Clinical characteristics of the patients

	Normal mfERG (*n* = 21)	Abnormal mfERG (*n* = 40)	*P*-Value
**Sex**			0.289^®^
Female	21 (36.8)	36 (63.2)	
Male	0 (0.0)	4 (100.0)	
**Disease** f (%)			0.486^©^
**SLE**	15 (37.5)	25 (62.5)	
**RA**	6 (28.6)	15 (71.4)	
**Age**			0.711^¶^
Mean ± SD	47.0 ± 11.94	46.7 ± 11.27	
Median [min, max]	48 [23,65]	46 [29,62]	
**Duration of Treatment** (years)			0.813^¶^
Mean ± SD	13.23 ± 5.83	13.15 ± 5.63	
Median [min, max]	13 [7,32]	11.5 [7,24]	
**Daily Dose (**mg/day**)**			0.850^¶^
Mean ± SD	202.50 ± 49.29	215.0 ± 65.09	
Median [min, max]	200 [150,400]	200 [150,400]	
**Total Dose of HCQ (mg)**			0.418^¶^
Mean ± SD	978.20 ± 523.20	1034.77 ± 590.09	
Median [min, max]	930.75 [511,2920]	590.09 [511,2920]	
**BCVA** (Best-corrected visual acuity) **Decimal**			0.782^¶^
Mean ± SD	0.88 ± 0.18	0.81 ± 0.27	
Median [min, max]	0.90 [0.2,1.0]	1.0 [0.2,1.0]	
**log MAR** [Logarithm of the Minimum Angle of Resolution]			0.172^¶^
Mean ± SD	0.069 ± 0.13	0.130 ± 0.20	
Median [min, max]	0.046 [0.0,0.07]	0.0 [0.0,0.07]	

¶: Resulted from independent t-test and *P* value < 0.05

©: Resulted from chi-square test

®: Resulted from Fisher's exact test

*SLE* Systemic lupus erythematosus, *RA* Rheumatoid arthritis, *HCQ* Hydroxychloroquine, *mfERG* Multifocal electroretinography

### SCP vessel density (%) parameters of OCTA

Mean SCP vessel density (%) parameters in Abnormal mfERG and Normal mfERG groups for all patients and also separately for SLE and RA subgroups are shown in Table 2. There was no significant difference between Abnormal mfERG and Normal mfERG groups in any of the SCP vessel density parameters, although in patients with RA the mean SCP vessel density (%) of Whole image, inferior Hemi, ParaFovea and PeriFovea in the Abnormal mfERG group were significantly lower than in the normal group (*P* -VALUE < 0.05).

**Table 2:** SCP vessel density (%) parameters of OCTA

Variables		SLE (***n*** = 40)	RA (***n*** = 21)	All patients (***n*** = 61)
Whole image	Normal mfERG	44.53 ± 4.84	50.68 ± 2.63	46.28 ± 5.08
	Abnormal mfERG	45.28 ± 4.60	44.71 ± 4.91	45.06 ± 4.66
	***P*-Value©**	0.627	0.019*	0.575
Superior Hemi	Normal mfERG	44.75 ± 4.72	50.00 ± 2.86	46.25 ± 4.80
	Abnormal mfERG	44.81 ± 4.68	45.00 ± 5.81	44.88 ± 5.06
	***P*-Value©**	0.972	0.085	0.549
Inferior Hemi	Normal mfERG	44.34 ± 5.04	51.22 ± 2.43	46.31 ± 5.66
	Abnormal mfERG	45.71 ± 4.58	45.21 ± 5.52	45.52 ± 4.88
	***P*-Value©**	0.397	0.032*	0.520
Fovea	Normal mfERG	13.27 ± 8.87	17.06 ± 3.61	14.35 ± 7.97
	Abnormal mfERG	13.66 ± 6.83	12.25 ± 8.36	13.13 ± 7.36
	***P*-Value©**	0.876	0.234	0.549
ParaFovea	Normal mfERG	43.78 ± 7.95	51.38 ± 4.21	45.95 ± 7.30
	Abnormal mfERG	46.01 ± 6.67	42.39 ± 6.72	44.76 ± 6.65
	***P*-Value©**	0.277	0.012*	0.626
PeriFovea	Normal mfERG	45.69 ± 5.45	51.98 ± 2.75	47.49 ± 5.54
	Abnormal mfERG	46.01 ± 4.52	46.41 ± 4.98	46.15 ± 4.62
	***P*-Value©**	0.845	0.030*	0.324

*P value < 0.05 and analysis using by independent t-test

*SLE* Systemic lupus erythematosus, *RA* Rheumatoid arthritis, *mfERG* Multifocal electroretinography, *OCTA* Optical coherence tomography angiography, *SCP* Superficial capillary plexus

### DCP vessel density (%) parameters of OCTA

Mean DCP vessel density (%) parameters in Abnormal mfERG and Normal mfERG groups for all patients and also separately for SLE and RA sub groups are shown in Table 3. There was a decrease in mean DCP vessel density (%) in the Abnormal mfERG group compared to the normal group in the areas of Whole image, Superior Hemi, Inferior Hemi, PeriFove, which is statistically significant. (*P*-VALUE < 0.05). This difference is also observed in the subgroup of people with RA, but no significant difference was observed in the SLE subgroup.

**Table 3:** DCP vessel density (%) parameters of OCTA and FAZ And Flow Area

Variables		SLE (***n*** = 40)	RA (***n*** = 21)	All patients (***n*** = 61)
Whole image	Normal mfERG	42.71 ± 10.59	53.76 ± 6.10	45.86 ± 10.60
	Abnormal mfERG	39.99 ± 7.46	40.44 ± 9.12	40.16 ± 8.17
	***P*-Value©**	0.347	0.009*	0.023*
Superior Hemi	Normal mfERG	42.31 ± 10.36	52.9 ± 5.36	45.33 ± 10.41
	Abnormal mfERG	39.81 ± 7.61	40.64 ± 9.11	40.12 ± 8.10
	***P*-Value©**	0.387	0.012*	0.034*
Inferior Hemi	Normal mfERG	43.07 ± 11.21	54.72 ± 6.71	46.40 ± 11.35
	Abnormal mfERG	40.16 ± 7.67	40.26 ± 10.30	40.20 ± 8.62
	***P*-Value©**	0.335	0.009*	0.020*
Fovea	Normal mfERG	29.67 ± 8.87	35.7 ± 5.87	31.39 ± 8.25
	Abnormal mfERG	30.61 ± 9.52	26.49 ± 12.52	29.07 ± 10.79
	***P*-Value©**	0.7590	0.133	0.447
ParaFovea	Normal mfERG	49.25 ± 9.92	57.96 ± 2.94	51.74 ± 9.32
	Abnormal mfERG	49.56 ± 6.70	46.24 ± 9.70	48.32 ± 8.01
	***P*-Value©**	0.906	0.018*	0.187
PeriFovea	Normal mfERG	42.71 ± 12.10	55.94 ± 6.60	46.44 ± 12.32
	Abnormal mfERG	39.88 ± 8.48	41.14 ± 10.3	40.36 ± 9.09
	***P*-Value©**	0.391	0.008*	0.031*
FAZ (.mm2)	Normal mfERG	0.38 ± 0.41	0.27 ± 0.05	0.35 ± 0.35
	Abnormal mfERG	0.36 ± 0.18	0.31 ± 0.18	0.34 ± 0.18
	***P*-Value©**	0.784	0.748	0.899
Flow Area of Outer Retina (mm^2^)	Normal mfERG	1.40 ± 0.58	0.93 ± 0. 52	1.27 ± 0.59
	Abnormal mfERG	1.17 ± 0.56	1.44 ± 0.46	1.27 ± 0.53
	***P*-Value©**	0.235	0.060	0.944
Flow Area of Choriocapillaris (mm^2^)	Normal mfERG	2.16 ± 0.11	2.27 ± 0.06	2.19 ± 0.11
	Abnormal mfERG	2.15 ± 0.12	2.08 ± 0.17	2.12 ± 0.15
	***P*-Value©**	0.693	0.002*	0.050^*^

*P value < 0.05 and analysis using by independent t-test

*SLE* Systemic lupus erythematosus, *RA* Rheumatoid arthritis, *mfERG* Multifocal electroretinography, *OCTA* Optical coherence tomography angiography, *DCP* Deep capillary plexus, *FAZ* Foveal avascular zone

### FAZ and FLOW area parameters of OCTA

Mean FAZ (mm^2^) and FLOW Area (mm^2^) Parameters in Abnormal mfERG and Normal mfERG groups for all patients and also separately for SLE and RA subgroups are shown in Table 3. The mean of none of the parameters of FAZ (mm^2^), Flow Area of Outer Retina (mm^2^) and Flow Area of Choriocapillaris (mm^2^) were not statistically significant between the groups Abnormal mfERG and Normal mfERG. (*p*-value> 0.05). However, the mean Flow Area of Choriocapillaris (mm^2^) in RA subgroup with Abnormal mfERG was statistically significantly lower than RA subgroup with Normal mfERG (*p* -value < 0.05).

Table 4 shows the relationship between Vessel Density, Retinal Thickness, FAZ, and Flow Area with the cumulative l dose of Hydroxychloroquine and the duration of treatment. The mean SCP Vessel Density in Whole Image and PeriFovea regions, as well as the mean DCP Vessel Density in Whole Image and PeriFovea regions showed a strong and inverse relationship with duration of treatment and cumulative dose, but the mean FAZ and Flow Area in Outer Retina and Choriocapillaris did not show a statistically significant relationship with drug duration or cumulative dose of drug.

**Table 4:** Pearson correlation between Duration of the treatment, Cumulative dose and Vessel Density, FAZ, Flow Area based

Variables	Duration of treatment (p)	Cumulative dose (p)
SCP vessel Density (%)		
Whole Image	−0.356 (0.005) ^*^	− 0.294 (0.021) ^*^
Fovea	0.064 (0.622)	−0.008 (0.952)
ParaFovea	−0.236 (0.067)	− 0.213 (0.099)
PeriFovea	−0.403 (0.001) ^*^	− 0.330 (0.009) ^*^
DCP Vessel Density (%)		
Whole Image	−0.272 (0.034) ^*^	− 0.278 (0.030) ^*^
Fovea	0.094 (0.469)	−0.21 (0.869)
ParaFovea	−0.177 (0.177)	− 0.181 (0.163)
PeriFovea	−0.272 (0.034) ^*^	− 0.274 (0.032) ^*^
FAZ (mm^2^)	−0.064 (0.627)	− 0.057 (0.664)
Flow Area of Outer Retina (mm^2^)	0.188 (0.147)	0.098 (0.451)
Flow Area of Choriocapillaris (mm^2^)	−0.206 (0.112)	−0.171 (0.187)

*: *P* value <0.05 and analysis using by pearson correlation test

*SCP* Superficial capillary plexus, *DCP* Deep capillary plexus, *FAZ* Foveal avascular zone, *HCQ* Hydroxychloroquine

## Discussion

Since early detection of HCQ-induced retinopathy is important, it is of great interest to find a modality that helps diagnose early-stage of retinopathy. In 2016, the AAO guideline proposed various modalities, including SD-OCT, AVF, FAF, and mfERG, for screening of HCQ-induced retinopathy, but none are gold standard [15]. The mfERG is more sensitive for diagnosis of HCQ-induced retinopathy in the early stages than the combination of AVF and SD-OCT tests [25, 29]. For this reason, in this study, based on mfERG changes, we divided patients into two groups: patients with Abnormal mfERG (Case group) and patients with Normal mfERG (Control group) and evaluated them with OCTA imaging. Patients with RA and SLE were also evaluated in separate subgroups. Because HCQ use for more than 5 years is a high risk for HCQ-induced retinopathy [15], we enrolled patients with at least 7 years of HCQ use.

Based on our findings, the mean Vessel Density (VD) in the SCP layer was not significantly different between the Abnormal mfERG and Normal mfERG groups, although in the RA subgroup, VD in Whole Image, ParaFovea and PeriFovea of the SCP layer in the Abnormal mfERG group decreased compared to the normal group. But it was not observed in SLE patients. The mean VD in the DCP layer (Whole Image, PeriFovea,) in the Abnormal mfERG group was lower than the normal group. This difference was also present in the RA subgroup but was not observed in the SLE subgroup. The mean VD in the Fovea area did not differ between the two groups. Also, FAZ Area and Flow Area (outer retina, choriocapillaris) were not different in the two groups and only Choriocapillaris Flow Area was lower in RA patients with Abnormal mfERG compared to RA patients with Normal mfERG.

To the best of our knowledge, limited studies have been conducted on the use of OCTA in the evaluation of HCQ-induced retinopathy, the results of which are also controversial. Some studies have evaluated OCTA changes between healthy individuals and patients with a history of HCQ use, and some studies have evaluated OCTA changes between High Risk (HCQ users over 5 years) and Low Risk (HCQ users less than 5 years) [22, 23, 28, 30–32]. A review of studies in this field shows confusing results. To our knowledge, no study has been performed to evaluate changes in OCTA among patients with HCQ-induced retinopathy based on mfERG and those without retinopathy.

In the study of Tarakcioglu et al., which was performed among 102 patients, No significant difference was observed between the HCQ treatment group and the control group in the parameters of FAZ, Flow Area, vascular density, SCP and DCP [28]. Based on our results, the patients in both groups were similar in the parameters of FAZ, Flow Area and SCP vascular density. In Bulut et al. study, the mean of vascular density (both SCP and DCP layers) in the group with HCQ use< 5 years was higher than the group with HCQ use > 5 years and also the FAZ Area in the group with HCQ use > 5 years was wider [22]; While in our study, vascular density in DCP was significantly different between the two groups, Their study was performed in patients with RA, Sjogren, SLE and Connective tissue disease, While our study was performed in SLE and RA patients; The duration of treatment and the cumulative dose of HCQ were also lower than in our study. In Goker et al., The mean VD of the HCQ group was lower than group of healthy individuals, and the FAZ Area was higher in the HCQ group than in healthy individuals [30]. In this study, patients were compared with healthy people. Since rheumatologic diseases, especially SLE, affect retinal vascularity [33], the results may not be related to HCQ. While we compared OCTA parameters in two groups of patients that can eliminate vascular changes caused by the disease itself.

In the Forte et al. study, was reported that with increasing cumulative dose and duration of HCQ use, VD in SCP and DCP decreased and also FAZ Area increased with Swept-source optical coherence tomography (SS-OCT) and separately, the vascular density in the areas of Fovea, PeriFovea, ParaFovea was not determined and the number of patients was 10 [23]. Based on our results with RTVue XR-OCTA, VD, especially in the PeriFovea area, is inversely related to the cumulative dose and duration of HCQ.

In our study, changes in vascular density, especially in the DCP layer in RA patients, differed between the two groups of Abnormal mfERG and Normal mfERG. The Telek et al. study also found that HCQ-induced retinopathy changes were more common in RA patients than in SLE patients. This may be due to longer use of HCQ or older patients [34]. Ozek et al. evaluated changes in OCTA in RA patients. VD in the DCP layer (Deep Temporal, Deep Hemi inferior) was lower in RA patients with a history of HCQ of more than 5 years compared to healthy individuals and also compared to individuals with a history of HCQ of less than 5 years [31].

In our study, we concluded that VD in the SCP layer (Whole Image, PeriFovea) was inversely related to the cumulative dose of the drug and the duration of drug administration. Also, VD in the DCP layer (Whole Image, PeriFovea) is inversely proportional to the duration of HCQ consumption. However, no relationship was observed between FAZ Area and Flow Area with drug duration and cumulative dose of HCQ. The mechanism of HCQ-induced retinal toxicity is not well understood. According to some studies, the accumulation of HCQ in RPE cells interferes with the clearance of intracellular toxic substances. That the inner layers and outer layers of the retina are damaged and some studies have suggested that the inner layer of the retina is not involved [6]. In the study of Kan et al. stated that ganglion cell-inner plexiform layer (GC-IPL) thinning occurs in patients with a history of HCQ before changes in AVF and RPE changes [35]. The study by Bulut et al. also showed that in the early stages of HCQ-induced retinal toxicity, the thickness of GC-IPL decreases. This reduction is related to the cumulative dose and duration of HCQ use [36]. In our study, DCP changes were greater than SCP, although these changes were not observed in SLE patients. In the study of De Sisternes et al., it was stated that HCQ retinopathy first begins in the photoreceptors and the paraFovea region, and that the Fovea is involved in more advanced stages [5]. In our study, FAZ Area and VD in Fovea Area in the two groups were not significantly different, which could be due to the fact that Fovea area is not involved in the early and subclinical stages of HCQ retinopathy. Flow area in Choriocapillaris was lower in RA patients with abnormal mfERG than in the other group, which may indicate that Choriocapillaris is involved in early HCQ retinopathy, but this difference was not observed in the SLE group, so further studies are needed.

The patients we evaluated all had normal AVF, FAF, SD-OCT, but some had evidence of early stage of HCQ-induced retinopathy at mfERG.

The strength of our study compared to other studies is that we performed OCTA in patients with a history of HCQ in two groups: Abnormal mfERG (with subclinical or early stage of HCQ Retinopathy) and Normal mfERG (without HCQ Retinopathy). To evaluate whether OCTA can help as a sensitive test to diagnose subclinical or early-stage retinopathy. Another feature of our study is that we separately evaluated RA and SLE patients who may have different immunological mechanisms. The limitations of our study are that it is cross-sectional and retrospective and the sample size is small, so doing a prospective study with more patients will help to get more accurate results. Also, the study of OCTA parameters in patients who have proven retinopathy due to HCQ in all three tests SD-OCT, mfERG, AVF can provide us with more useful information. But usually, the number of these patients is small.

In conclusion, we found that the density of retinal vessels in the Perifovea area decreased with increasing cumulative dose of and duration of treatment, but the FAZ Area did not change significantly. In RA patients with Abnormal mfERG (early stage of HCQ Retinopathy) mean VD of PeriFovea, ParaFovea area of DCP layer was reduced compared to RA patients with Normal mfERG (without HCQ Retinopathy) but FAZ Area and VD of Fovea did not change significantly in early retinopathy. OCTA may be used as a non-invasive modality to diagnose early stages of HCQ retinopathy but our knowledge in this area is still limited and more studies are needed.

## Data Availability

The datasets used and/or analyzed during the current study are available from the corresponding author on reasonable request.

## Abbreviations

SLE: Systemic lupus erythematosus
RA: Rheumatoid arthritis
HCQ: Hydroxychloroquine
mfERG: Multifocal electroretinography
OCTA: Optical coherence tomography angiography
SCP: Superficial capillary plexus
DCP: Deep capillary plexus
FAZ: Foveal avascular zone
